# Analysis of Experts’ Quantitative Assessment of Adolescent Basketball Players and the Role of Anthropometric and Physiological Attributes

**DOI:** 10.2478/hukin-2014-0080

**Published:** 2014-10-10

**Authors:** Erik Štrumbelj, Frane Erčulj

**Affiliations:** 1 Faculty of Computer and Information Science, University of Ljubljana, Ljubljana, Slovenia.; 2 Faculty of Sport, University of Ljubljana.

**Keywords:** sports, coaching, morphology, motor skills, performance evaluation, players’ selection

## Abstract

In this paper, we investigated two questions: (1) can measurements of anthropometric and physiological attributes substitute for expert assessment of adolescent basketball players, and (2) how much does the quantitative assessment of a player vary among experts? The first question is relevant to the potential simplification of the player selection process. The second question pertains directly to the validity of expert quantitative assessment. Our research was based on data from 148 U14 female and male basketball players. For each player, an array of anthropometric and physiological attributes was recorded, including body height, body mass, BMI, and several motor skill tests. Furthermore, each player’s current ability and potential ability were quantitatively evaluated by two different experts from a group of seven experts. Analysis of the recorded data showed that the anthropometric and physiological attributes explained between 15% and 40% of the variance in experts’ scores. The primary predictive attributes were speed and agility (for predicting current ability) and body height and growth potential (for predicting potential ability). We concluded that these attributes were not sufficiently informative to act as a substitute for expert assessment of the players’ current or potential ability. There is substantial variability in different experts’ scores of the same player’s ability. However, the differences between experts are mostly in scale, and the relationships between experts’ scores are monotonic. That is, different experts rank players on ability very similarly, but their scores are not well calibrated.

## Introduction

Athlete selection – selecting a subset of the most talented athletes for competition or special training from a larger group of potential candidates – is an important process in sports. Basketball players participate in selection processes at several different stages of their career at the club, regional, and national levels. An effective selection program is a vital component of selecting the national basketball team. National selection programs can also be used to monitor and evaluate the quality of work at the club level and can lead to more focused training of selected younger athletes with senior-level potential.

Athlete selection involves evaluating the current performance of athletes and/or predicting their future performance. Various criteria are used when selecting the best basketball players. The most important and most frequently used criterion is the quality of past performances during competition. However, this cannot be the only criterion, especially when considering adolescent players. While assessing the potential of youth players with exceptional success in competition is simple, most youth players, due to their age and lack of experience, have not yet developed and/or are unable to fully display their abilities in a competition setting (Baechle and Earle, 2008). Therefore, there are benefits of considering other indicators of current and potential ability of youth basketball players.

In basketball, additional indices typically include anthropometric, physiological, technical, tactical, and psychosocial attributes and potential interactions between them. As such, the problem of selection is a complex task, and measurement of all relevant attributes is often impractical or impossible. Athlete selection usually falls on basketball experts to perform the evaluation based on their expert knowledge and experience. Within a competition, a player’s play time can also be used as an indicator of the player’s ability. Hoffman et al. (1996) showed that the coach’s evaluation explained 56% to 86% of the playing time variance for 29 male Div. I college basketball players over 4 years. Therefore, we presumed that expert scores were the best available evaluation method for a player’s actual and potential ability. Furthermore, understanding how experts evaluate basketball players is crucial to understanding and improving the selection process. In this research, we focused on two questions:

- Do different experts rate adolescent basketball players in a substantially different way, and
- how much of the variability in experts’ scores is explained with easy-to-measure anthropometric and physiological attributes?

The first question is fundamental to validate the use of quantitative expert assessment as an objective assessment of a player’s current or future ability. We hypothesized that basketball experts would score the same player very similarly. Regarding the second question, we hypothesized that a relationship existed between a player’s attributes and experts’ assessment of that player’s skill. If the amount of explained variance is high, it would eliminate, at least in part, the need for expert assessment, which could lead to simplification of the selection process.

To our knowledge, no study has compared experts’ evaluations of basketball players. However, there are several studies that have investigated the relationships between basketball players’ anthropometric/physiological characteristics, sport specific skills and expert evaluation. Although in most cases, expert evaluation was assessed indirectly, by comparing study participants with a pre-selected, elite group of players or using players’ play time in competition as a main variable considered in expert evaluation.

Delextrat and Cohen (2008) compared two groups of 8 players: a university team competing in the top-tier British Universities competition (elite-level players) and a university team competing in a lower-tier university competition (average-level players). Their results suggest that the main differences between the two groups were in agility and anaerobic power. Similarly, Torres-Unda et al. (2013) compared a group of 16 elite 13–14 year-old basketball players to 46 other players from the region. On average, the elite players outperformed the other players in jumping, endurance, speed and special agility tests.

Other relevant studies include the work by Coelho E Silva et al. (2008), who investigated the effects of size and maturity on basketball players’ functional capacities and sport-specific skills. The two predictors explained little variance in functional capacities and a low to moderate amount of variance in basketball-specific skills. Ben Abdelkrim et al. (2010) also concluded in their study of competitive-level differences that basketball players should have good intermittent aerobic endurance and agility. Several other studies have also suggested the importance of agility in basketball players (Erčulj, 2005; Balčiunas et al., 2006; Karpowicz, 2006; Litkowycz et al., 2008; Alemdaroglu, 2012; Jakovljević et al., 2012).

Therefore, most of the related research is limited to either observing the correlation between player characteristics and expert evaluations or finding significant differences between average and elite players. These results show which characteristics might be relevant for selecting players, but we do not know how much of the variance we can explain due to the correlation between explanatory variables. More relevant research regarding the ability to predict basketball players’ ability is less common. In a large sample of Australian U16 championship players, Hoare (2000) showed that anthropometric and physiological attributes explained a significant proportion of variance in post hoc expert evaluation of competition performance. Hoffman et al. (1996) found that leg strength, vertical jump, speed, and agility explained 64 to 81% of the variance in playing time.

## Methods

### Participants

Our sample was composed of 62 youth female and 86 youth male basketball players. Their anthropometric and physiological characteristics were measured in 2011 when they voluntarily participated in the annual regional selection performed by the Basketball Federation of Slovenia. All measurements were performed on a single day in December, at the end of the youth competitive season. The main purpose of this selection program was a preliminary selection of a broader group of potential candidates for the U15 national teams. The male participants were all 13 years old, with an average of 5.4 years of basketball experience. The female participants were composed of 12-year-olds (N = 49) and 13-year-olds (N = 13), with an average of 3.8 years of basketball experience. Note that 12 of the 148 participants were later selected for the U15 national team.

### Measures and procedures

Measurements are summarized in Table 1. A bioelectrical impedance-based body-fat analyzer (TANITA MC-980 MA, Japan) was used to estimate body fat. A detailed description of the measurement procedures for motor skill tests (S20, V20, TSS, VSS, CMJ, dCMJH) can be found in Erčulj and Bračič (2007).

Note that the 30–15IFT endurance test (Buchheit, 2010) was developed for testing handball endurance. For basketball-specific endurance, the running distance was shortened from 40 to 20 m, and the corresponding parameters were appropriately modified. This modification for basketball was previously validated (Erčulj et al., 2012).

The maximal achieved velocity was used to estimate maximal oxygen uptake (Buchheit, 2010):

$$\begin{array}{l} {\mathrm{VO}}_{2\max}(\mathrm{ml}/\min/\mathrm{kg})=28.3-2.15\times G-0.741\times A-\\ 0.0357\times P+0.0586\times A\times V+1.03\times V, \end{array}$$

where G was the participant’s gender (1 = male, 2 = female); and A, P, and V were the participant’s age, body mass, and maximum velocity achieved during the test, respectively.

### Expert assessment

The head of the national selection program and 7 regional basketball experts scored the participants’ current and potential ability. The head of the national selection program scored all the players, while each of the regional experts only assessed a subset of players from his or her corresponding region with whom he/she was familiar.

Experts were asked to score each player’s current ability and potential ability on a scale from 0.0 (worst) to 5.0 (best), and rough guidelines were provided to ensure that the experts’ scores were approximately on the same scale (Table 2). Experts made their assessment based on their knowledge and familiarity with the players.

### Analysis

Male and female subjects were analyzed separately. The regional experts’ scores were compared to the score given by the head of the selection program. We tested the hypothesis of equality of means via a t-test. To verify the extent to which the differences between the experts could be attributed to poor calibration, we applied isotonic regression to calibrate each regional expert’s scores to the head’s scores, while preserving the ranking. We reported strength of the relationships between variables in terms of Pearson correlation coefficients.

Before performing regression analysis, we removed highly correlated explanatory variables. Correlations between all explanatory variables and the target variables were reported. We used a linear model to evaluate the relationship between the morphological/motor factors and the head of the selection program’s scores. We applied backward elimination for factor subset selection and used the Bayesian information criterion (BIC) to avoid over fitting the data (and to obtain a more conservative R^2). The procedure was performed separately for the participants’ current ability scores and the difference between current and potential ability scores (potential – current), giving a total of 4 linear models. All statistical computation was performed in R.

## Results

Table 3 summarizes the measurements and compares the data between male and female subjects. The male youth players were significantly taller, had a longer wingspan, and performed better in all tests compared to the female players. Therefore, the female youth players had significantly higher body fat and body fat percentage compared to the male players.

A summary of experts’ scores is given in Table 4. Some, but not all, regional experts’ mean scores were significantly different from the program head’s mean score on the same subset of participants. The correlation coefficients between the two scores were moderate to high, with the lowest correlation coefficient being 0.70. After calibrating each regional expert’s scores to the head expert’s scores, without changing the order in which they scored the participants, all regional experts’ scores correlated highly with the head’s scores.

Correlation coefficients between all the explanatory variables and the target variables are shown in Table 5. Before further regression analysis, we removed the following redundant predictors for both the female and male youth basketball players:

- Due to strong correlation with the BMI, we removed BODY MASS, FAT%, and FATkg (r > 0.80).
- We kept V20 (VSS) and removed S20 (V20) (all r > 0.75).
- We kept HEIGHT and removed SPAN (r > 0.90).

The selected models of the current and the potential - current target variables (for male and female subjects separately) are shown in Table 6. In both cases, we explained a larger proportion of the variance in the target variable for the female players. For the female subjects, the amount of variance explained by the explanatory variables was moderate, and for the male subjects it was low.

The explanatory variables in each of the models for male subjects were a subset of the explanatory variables for the corresponding model for female subjects. V20 was a significant variable for the current model for both female and male youth basketball players. Additionally, HEIGHT and VO_2max_ also played a significant role for the female players. For the potential – current model, HEIGHT and the BMI were significant variables for both female and male youth basketball players. Additionally, YTRAIN also played a significant role for the female subjects.

## Discussion

The result showing that male youth basketball players outperformed female players is consistent with similar studies on the general population (McCarthy et al., 2006) and basketball players (Jakovljević et al., 2011a; Jakovljević et al., 2011b). Also noteworthy is that the BMI of our participants was similar to that of the general population (McCarthy et al., 2006).

Our results showing a high correlation between sprinting (S20) and dribbling (V20) are consistent with other studies (Meckel et al., 2009). However, it is worth noting that this outcome was expected for our sample of trained youth basketball players. For samples of less trained or untrained players, we would expect the differences to be larger, and including both predictors would be worthy. The same conclusion applies to sprinting (TSS) and dribbling (VSS) with changes of direction.

Our findings of a high correlation between CMJ and dCMJH are also in line with the results of previous studies. Erčulj (2005) studied 16- and 17-year-old male basketball players and found a high correlation between CMJ and dCMJH and a 21.5% increase in mean jump height when the use of hands was allowed during the jump. We observed a similar 4.97 cm (20.0%) increase in jump height for male participants and a 3.56 cm (17.3%) increase in jump height for female participants when the use of hands was allowed during the jump. A small 1.5% difference between the results of male participants of our study and those of the Erčulj’s study could easily be attributed to the age difference of the study participants and a more selected sample used by Erčulj (2005).

### Experts’ scores

In several cases, the regional expert’s mean score was significantly different from the head expert’s scores (Table 4). However, in all cases, a strong linear relationship existed between the experts’ scores, even before isotonic regression calibration. While the scales used by the experts might not always be the same, despite our attempt to enforce a common scale, the relationships between their scores were monotonic.

These results suggest that experts were proficient at ranking players from best to worst but were poor at scoring players in the absolute (that is, assigning a number that says how well a player compares to other players). This phenomenon should be taken into account in any study that uses or compares quantitative assessments from different experts. Expert scores should be checked for calibration and recalibrated to the same scale, if necessary.

### Models

Our data are consistent with other studies showing that players’ score pertaining to agility and sprinting correlate with playing quality and, therefore, expert scores (Erčulj, 2005; Balčiunas et al., 2006; Karpowicz, 2006; Litkowycz et al., 2008; Alemdaroglu, 2012; Jakovljević et al., 2012). Although these and several other explanatory variables correlate with experts’ scores (Table 5), only a few variables are sufficient to maximize the amount of explained variance. Therefore, in terms of predicting experts’ scores, most of the used explanatory variables are redundant.

Body height and body-fat percentage appear in the model of the difference between the potential and current score for both male and female subjects. Both variables are connected to body height. Current height is a direct indicator of potential height and body fat is an indicator of growth potential, with lower body fat indicating higher growth potential. Body-fat percentage can be an indicator of biological maturity, which correlates with skill tests that require strength and speed (Malina et al., 2004). Therefore, body fat can indirectly correlate with current basketball performance. However, on its own, body fat has a negative effect on current basketball performance (Erčulj and Bračič, 2010). Lower body-fat percentage is an indicator of larger growth potential and, therefore, more potential of playing senior-level professional basketball (Erčulj and Bračič, 2010). Increased body-fat percentage is also known to decrease the frequency and duration of growth hormone secretion (Grumbach and Styne, 2003). In such individuals, we can expect a decrease or stop in height growth.

Therefore, for both male and female subjects, current and/or potential height is the main explanatory variable for playing potential (relative to current score). Competitive basketball at a high level demands individuals with (at a minimum) above-average body height, and players playing the role of forwards or centers have to be of extreme body height. Body height cannot be improved by training, and lack of height can be compensated for by other characteristics, but only to a certain extent.

The proportion of variance in expert scores that can be explained by the anthropometric and physiological attributes that we determined is similar to that reported by Hoare (2000). Hoffman et al. (1996) reported higher proportions. However, a smaller sample of players was used in their study, and the target variable was the amount of playing time, and not expert scores.

In conclusion, the proportion of the explained variance is significant; however, it is not large enough to be a good predictive model of expert scores. Therefore, the array of attributes used in this study is insufficient to substitute for expert quantitative assessment. More research is needed, especially including basketball-specific skills, competitive performance indicators, and psychosocial components.

## Figures and Tables

**Table 1:** Brief description of measurements

Abbrv.	Description
HEIGHT	Body height [cm].
BM	Body mass [kg].
BMI	Body mass index.
SPAN	Wingspan [cm].
FAT%	Body fat percentage.
FATkg	Body fat [kg].
S20	20 meter sprint [s].
V20	20 meter dribble [s].
TSS	Sprint with change of direction 6 times 5 meters [s].
VSS	Dribble with change of direction 6 times 5 meters [s].
CMJ	Countermovement jump height [cm].
dCMJH	Countermovement jump height, with use of arms [cm].
VO_2max_	Maximal oxygen uptake estimated by 30–15 IFT test [ml/min/kg].
YTRAIN	Training experience (at club level) [years].

**Table 2:** Guidelines for scoring

Current ability:

4.0 – 5.0: top of the participants
3.0 – 3.9: above-average ability
2.0 – 2.9: average ability
1.0 – 1.9: below-average ability
0 – 0.9: bottom of the participants
Potential ability:

4.0 – 5.0: potential for highest tier of European competitions
3.0 – 3.9: potential for first-tier senior-level national competitions
2.0 – 2.9: potential for second-tier senior-level national competitions
1.0 – 1.9: low potential for senior-level competitions
0 – 0.9: no potential for senior-level competitions

**Table 3:** Means and standard deviations (s) of measurements

	Female		Male		

	Mean	s	Mean	s	p
HEIGHT	166.13	7.41	172.82	8.88	< 10^−5^
BM	57.27	12.29	59.93	12.30	0.19
BMI	20.59	3.16	19.88	2.64	0.15
SPAN	168.48	8.08	177.03	11.14	< 10^−5^
FAT%	24.06	4.27	16.03	3.51	< 10^−5^
FATkg	14.19	5.56	9.89	3.95	< 10^−5^
S20	3.67	0.21	3.47	0.19	< 10^−5^
V20	4.01	0.23	3.71	0.23	< 10^−5^
TSS	9.77	0.55	9.00	0.78	< 10^−5^
VSS	10.40	0.62	9.43	1.08	< 10^−5^
CMJ	20.52	3.91	24.90	4.49	< 10^−5^
dCMJH	3.56	2.48	4.97	2.05	< 10^−5^
VO_2max_	41.25	2.41	43.73	2.52	< 10^−5^

YTRAIN	4.03	2.10	5.40	1.88	< 10^−5^

**Table 4:** Experts’ mean score and standard deviation (s) compared to head’s mean score and standard deviation (s_head_), p-value for equality of means (p), correlation coefficient before (r) and after calibration (r’), and number of participants in subset (N). The data are broken down by gender, score type, and expert

Subset			Mean	s	Mean_head_	s_head_	p	r	r′	N
Female youth basketball players	Current	E_head_	3.28	0.46	-	-	-	-	-	62
		E_A_	2.51	1.18	3.46	0.39	3.93 × 10^−4^	0.92	0.97	16
		E_B_	3.47	1.06	3.19	0.42	1.98 × 10^−1^	0.70	0.90	16
		E_C_	1.89	0.71	3.15	0.54	2.41 × 10^−9^	0.90	0.95	14
		E_D_	3.28	1.06	3.30	0.53	8.92 × 10^−1^	0.90	0.97	12

	Potential	E_head_	3.70	0.46	-	-	-	-	-	62
		E_A_	2.39	0.86	3.91	0.29	1.50 × 10^−7^	0.79	0.96	16
		E_B_	3.51	0.68	3.62	0.46	3.61 × 10^−1^	0.72	0.90	16
		E_C_	2.61	0.84	3.56	0.55	9.98 × 10^−6^	0.81	0.89	14
		E_D_	3.38	1.21	3.64	0.55	2.38 × 10^−1^	0.94	0.99	12
	
Male youth basketball players	Current	E_head_	3.52	0.45	-	-	-	-	-	86
		E_E_	2.67	1.11	3.49	0.36	3.63 × 10^−4^	0.74	0.91	21
		E_F_	3.39	0.94	3.49	0.67	4.40 × 10^−1^	0.81	0.94	19
		E_G_	3.44	0.49	3.52	0.44	7.38 × 10^−2^	0.92	0.98	24

	Potential	E_head_	3.80	0.44	-	-	-	-	-	86
		E_E_	3.10	1.14	3.77	0.41	1.66 × 10^−3^	0.80	0.91	21
		E_F_	3.61	0.81	3.67	0.58	5.69 × 10^−1^	0.77	0.94	19
		E_G_	3.83	0.48	3.81	0.46	7.56 × 10^−1^	0.85	0.96	24

**Table 5:** Correlation coefficients between the measurements and the head expert’s scores

	Female subjects		Male subjects	

	Current	Potential - Current	Current	Potential - Current
HEIGHT	0.27^*^	0.21^§^	0.19^§^	0.19^§^
BM	0.25^*^	−0.13	−0.03^*^	−0.13
BMI	0.17	−0.35^**^	−0.14	−0.29^*^
SPAN	0.27^*^	0.12	0.21^§^	0.15
FAT%	−0.11	−0.37^***^	−0.11	−0.34^**^
FATkg	0.06	−0.29^**^	−0.8	−0.24^*^
S20	−0.46^***^	−0.11	−0.32^*^	−0.06
V20	−0.44^***^	−0.01	−0.44^**^	−0.07
TSS	−0.41^**^	0.02	−0.43^**^	0.07
VSS	−0.49^***^	0.03	−0.50^***^	0.12
CMJ	0.29^*^	−0.07	0.18^§^	0.12
CMJH	0.21^**^	0.02	0.20	0.10
VO_2max_	0.21	−0.01	0.46^***^	0.06

YTRAIN	0.03	−0.17^§^	0.23^§^	−0.47^**^

§ significant at the 0.15 level,

* significant at the 0.05 level,

** significant at the 0.01 level,

*** significant at the 0.001 level

**Table 6:** Summary of selected predictors and estimated coefficients for the linear models of experts’ scores

Model	Predictor	Coefficient	R^2
	constant	−1.505	**0.3899**
Female subjects (Current)	HEIGHT	0.0263^***^	
	V20	−0.6368^***^	
	VO_2max_	0.0725^***^	
	constant	−0.3954	**0.3926**
Female subjects (Potential – Current)	HEIGHT	0.0087^***^	
	BMI	−0.0241^***^	
	YTRAIN	−0.0384^***^	
Male subjects (Current)	constant	−6.3557	**0.1504**
	V20	−0.7620^***^	
Male subjects(Potential – Current)	constant	−0.8882^***^	**0.3573**
	HEIGHT	0.0117^***^	
	BMI	−0.0431^***^	

^***^ *significant at the 0.001 level.*

All four models were significant at the 0.001 level. When all predictors were kept in the model, we obtained R^2^ coefficients of 0.4556, 0.4645, 0.2627, and 0.4080 for the female subjects (Current), female subjects (Potential – Current), male subjects (Current), and male subjects (Potential – Current) target variables, respectively.
